# Genetic Variants at PRKCG Splice and UTR Sites Promote Cancer Susceptibility by Disrupting Epigenetic and miRNA Regulatory Network

**DOI:** 10.7150/jca.100911

**Published:** 2024-10-28

**Authors:** Fizzah Abid, Khushbukhat Khan, Naeem Mahmood Ashraf, Yasmin Badshah, Maria Shabbir, Janeen H Trembley, Tayyaba Afsar, Ali Almajwal, Suhail Razak

**Affiliations:** 1 Atta-ur-Rahman School of Applied Biosciences, National University of Sciences and Technology, Islamabad, 44000, Pakistan.; 2 School of Biochemistry and Biotechnology, University of the Punjab, Lahore, 54590, Pakistan.; 3 Minneapolis VA Health Care System Research Service, Minneapolis, MN, 55111 USA.; 4 Department of Laboratory Medicine and Pathology, University of Minnesota, Minneapolis, MN,55405 USA.; 5 Masonic Cancer Center, University of Minnesota, Minneapolis, MN,55407 USA.; 6 Department of Community Health Sciences, College of Applied Medical Sciences, King Saud University, Riyadh 11421, Saudi Arabia.

**Keywords:** database, *in-silico* tools, non-coding variants, PRKCG, splice sites, 3′UTR, 5′UTR

## Abstract

The changes in the protein kinase C gamma gene (PRKCG) expression are associated with both coding and non-coding variants. No studies have specifically established the association between PRKCG 3′UTR, 5′UTR, donor and acceptor splice variants with post-transcriptional changes through utilizing *in-silico* tools. The current study intends to uncover this linkage. In total, 419 3′ and 5′UTR variants were retrieved. 325 of these variant IDs were annotated as functionally significant. 18 variants impacted the transcription factors binding and therefore influenced the post-transcriptional regulatory activity while 7 variants affected regulatory mechanisms through histone modifications. 2 rsIDs (rs373228, rs446795) potentially impacted the interactions with RNA binding proteins. In addition to that, PRKCG showed high expression in brain cells and had variable expression in TCGA tumors, respectively. Furthermore, 5 3′ UTR variants were identified to be targeted by miRNAs. In total, 5 of these miRNAs (hsa-miR-663a, hsa-miR-324-5p, hsa-miR-646, hsa-miR-1205 and hsa-miR-4270) that targeted 3′UTRs (rs57483118, rs181418157 and rs60891969) showed differential expressions in distinct cancer types. The presence of 3′UTR variants likely altered the secondary structure of mRNA. The 7 rsIDs at 3′ UTR site caused the loss of function of authentic splice site at 10 positions was noted; at 1 position, gain of function was observed while at 2 positions no effect was identified. Moreover, the loss of donor and acceptor splice site was evident. Our results highlight the importance of non-coding regions that might boost our research capacity to predict and construct targeted therapeutic approaches.

## Introduction

The human genome contains about ten million SNPs (Single Nucleotide Polymorphisms) located in the non-coding and coding region of genes. Within the human genome, SNPs are the most abundant type of polymorphism identified. Genetic polymorphisms are regarded as the alternative forms of two or more alleles that result in distinct phenotypes. Moreover, SNPs occur approximately every 1000 base pairs on average across the genome 1. SNPs are categorized as functional and neutral. SNPs that confer disease onset through affecting phenotype are functional SNPs while neutral SNPs bring no deleterious effect to the subsequent translated protein 2. Reportedly, the frequency of SNPs in non-coding regions compared to coding regions is higher 3, 4. Through GWAS (Genome Wide Association Studies), it has been determined that non-coding regions constitute about 88% of SNPs linked with phenotype associated alterations, among which 45% occur in intronic while 43% reside in intergenic regions 5.

To add to this, the majority of the functional SNPs lie in the consensus sequences that separate exon and intron boundaries 6. SNPs in these conserved regions form splice junctions and generate splice variants that can alter the phenotypic expression 6. A polymorphism is also a mutation type, however, not all mutations come under the category of polymorphisms. The frequency of polymorphism is 1% or higher within a population in comparison to mutations that are rather rare and have a frequency of less than 1% 1. Furthermore, mutations can give rise to polymorphisms 7. During the evolutionary process, the mutation that has occurred at some time in the ancestral sequence may become polymorphism later on or get repaired or further mutated 8. Under the terminology “Genetic variations”, common polymorphisms or rare mutations can be described 1. In fact, for many genes, genomic variations, which affect the splicing process may represent up to 50% of all mutations that lead to gene dysfunction 9. Therefore, elucidation of the relationships between genetic variants, altered splicing patterns, post-transcriptional processes, and the resultant phenotypic changes is needed to understand disease onset 10, 11.

Approximately 3.7% of the variants are located in the UTRs (Untranslated Regions) according to GWAS 12. UTRs form non-coding transcript segments that surround the coding portion of a mRNA (messenger RNA) at the 5' and 3' ends 13. Interestingly, polymorphisms are reported in 3' UTRs while mutations dominate in the 5' UTRs 12. UTR region alterations disrupt transcriptional processes, mRNA stability, mRNA folding and translational processes. As 5' UTR sites are critical for recruiting ribosomes, variants at these sites can perturb protein synthesis pathways 14. To add to this, variants identified in 3' UTRs impact the interaction with miRNAs (microRNAs). Possibly, miRNAs are encoded by 4% of the genome and regulate more than 30% of the genes 15, 16.

The current study is centered on PRKCG, which belongs to the conventional class of PKCs 17. Variants in PRKCG gene lead to deleterious outcomes. Specifically, PRKCG gene is well-researched in relation to brain diseases. The presence of PRKCG variants disrupt neuronal signaling and leads to neuronal dysfunction 17. The role of PRKCG in osteosarcoma 18, colon cancer 19, gliomas 20, breast cancer 21, ovarian cancer 22 and hepatocellular carcinoma 23, 24 is established. However, not enough literature is available to elucidate its role in other cancer types. It acts as an oncogene and fosters tumor growth and facilitates metastasis 18. Its non-coding variants can increase cancer risk, but only a few studies have been conducted in this regard.

Moreover, with the increase in the number of genetic variants in databases, it becomes more challenging to determine their functional contributions in disease development through experimental approaches. Bioinformatics tools have eased the complexity of analyzing data based on variants. Before wet lab validation, *in-silico* findings can aid in narrowing down deleterious variants for screening of genetic diseases 25. The pace and accuracy of data processing have significantly accelerated with the employment of these tools in research 26. There is a gap in published information utilizing *in-silico* approaches to decipher the impact of these PRKCG variants on disease pathogenesis. We linked phenotypic variations associated with non-coding variants with gene expression, regulatory elements activity and chromatin accessibility due to disrupted transcription factors (TF) binding. Furthermore, we examined the linkage disequilibrium (LD) between the studied UTR variants as well as the impact of the variants on PRKCG functionality and structure.

## Methods

### Collection of Data

The data related to PRKCG 3′ UTR, 5′ UTR, splice donor and splice acceptor variants was fetched from Ensembl genome browser that provides open access to annotated datasets 27. Of the provided transcript IDs, the MANE transcript ENST00000263431.4 with 3149 base pairs and 697 amino acids was chosen. The information regarding COSMIC variants was retrieved from COSMIC, which is a curated database that provides access to the clinical data related to cancer and somatic variants 28.

### Processing of Retrieved Data

The IDs corresponding to 3′ and 5′ UTRs were input in RegulomeDB software 29 to filter out UTR variants with valid functional scores from 1-6. The RegulomeDB software uses manual annotations, and computational tools along with the experimental datasets from ENCODE 30 to identify putative regulatory variants. The closer the RegulomeDB score is to 1, the higher is the confidence of the variant having a significant functional impact, meaning that the variant presence could affect transcription factor binding and therefore the downstream mechanisms of transcription and translation. The RegulomeDB score of 2 also affects binding but as the scores shifts to 3, the chance variant site to affect transcription factor binding is less likely. Between the scores of 4-6, the experimental evidence of the variant affect binding is minimal 31.

### Regulatory Function and Tissue Expression Characterization

The variants that were identified through RegulomeDB were entered in rSNPbase3.1 software, HaploReg software, RBP-Var2 database and Gtex software. The rSNPbase3.1 software helped identify variants with proximal and distal post-transcriptional regulatory activity along with their possibly regulated genes 32. The HaploReg software searched whether the 3′ and 5′ UTR variants were in linkage disequilibrium (LD) or not by utilizing data on chromatin state, regulatory changes, and conservation alteration 33. The RBP-Var2 database identified variants that affect RNA binding pattern and post-transcriptional interaction through miRNAs 34. The effect of variants on riboSNitches, which are regulatory elements present within the untranslated regions of mRNA and have functions comparable to riboswitch 35, was determined from RBP-Var2 database. The relationship between 3′ and 5′ UTR variants and PRKCG gene expression across multiple human tissues was found out through Gtex software 36 while the UALCAN web resource 37 was used for ascertaining PRKCG expression in TCGA (The *Cancer Genome Atlas)* cancers 38, 39. The web resource provides RNA-sequencing data from the TCGA for gene expression analysis. The brief data related to number of patients and normal individuals or clinical parameters, including the source of the sample is provided by UALCAN. To add to this, UALCAN does not use the exact cut-off value. The p-values that are less than 0.05 are considered statistically significant. Transcript Per Million (TPM) values that are greater than 1 indicate Median expression.

### Influence of Variants on miRNA Regulation

Our study focused on analyzing 3′ UTRs that are majorly targeted by miRNAs. Although 5′UTRs are targets of miRNAs their impact is less than that of 3′ UTR sites 40. Of the total 5′ and 3′ UTR variants, 3′ UTR variants were selected for further analysis. miRdSNP database 41 was explored to determine the miRNA targets of these 3′ UTR variants. Apart from miRdSNP database, information regarding 3′ UTR variants (that are targets of miRNAs) was retrieved from PolymiRTS Database 3.0 42, 43. The expression of these miRNAs in different cancers was discovered through miRCancer Database 44. The miRNA expression upregulation and downregulation is influenced by epigenetic changes that lead to changes on the histones marks and cause DNA unwrapping or wrapping around histones 45. To add to this, hypo and hypermethylation and the presence of variants in the promotor also impact miRNA gene expression 46, 47. Furthermore, miRNAs can act as oncogenes or as tumor suppressors and their aberrant expression levels contribute to oncogenesis. Moreover, because of 3′ UTR variants, the interaction of mRNA with miRNA also gets disrupted which leads to targeted protein over-expression 48. Our study tried to determine the effect of epigenetic alterations and variants' presence on PRKCG expression at the genetic level. The changed secondary structures because of 3′ UTR variants that act as miRNA target sites were analyzed using RNAfold. The software depicts the Minimum Free Energy (MFE) structure of RNA and variants' presence on base-pairing interactions and RNA stability 49, 50.

### Splice Site Identification

Genetic variations that are caused by mutations come into focus every year. Moreover, a larger proportion affects mRNA splicing give rise to altered structure of proteins 10. The ability of a particular sequence motif to function as a donor or acceptor splice site was identified using the SpliceAI software (https://spliceailookup.broadinstitute.org/) and MaxEntScan software (https://varseak.bio/). The scores presented by SpliceAI vary from 0-1 with higher scores indicating the loss of donor or acceptor site while scores near 0 indicate no effect of the variant on the splice site 51. The MaxENTScan scores also provide information related to the functionality of donor or acceptor sites. The data regarding the loss of function of the authentic splice site or whether the base substitution does not affect the splice site was gained through MaxEntScan 52.

### Graphical Representation

The results were plotted with the aid of Microsoft Excel 53. Column and Bar chart graph format was selected for representation of data. This chart format is particularly useful for comparing results of different software. For customization of obtained graphs, provided features in the Toolbar were accessed.

## Results

### Processing and Analysis of Collected Data

**Figure 1:**
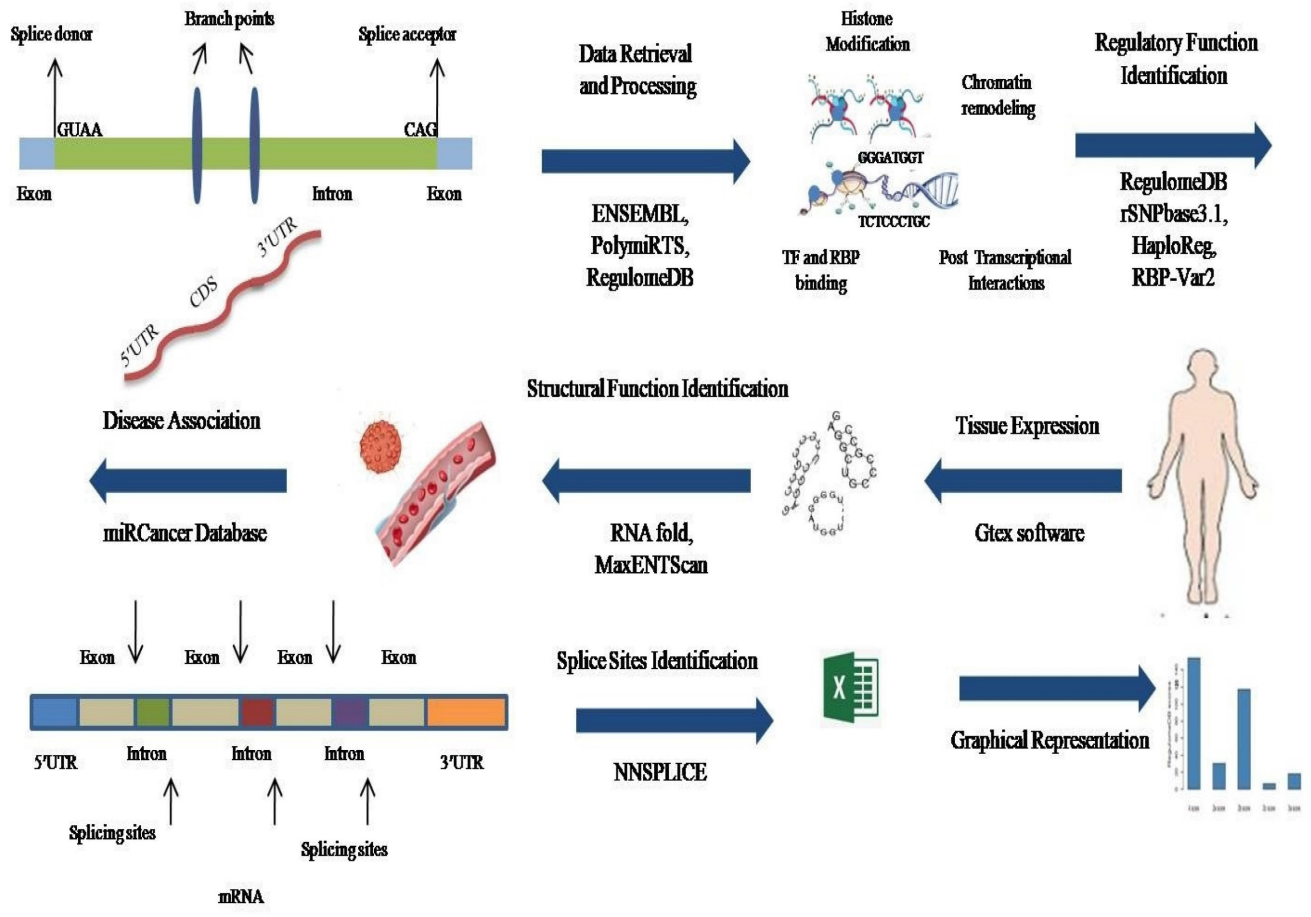
Overview of the research study—representation through schematic diagram.

**Figure 2:**
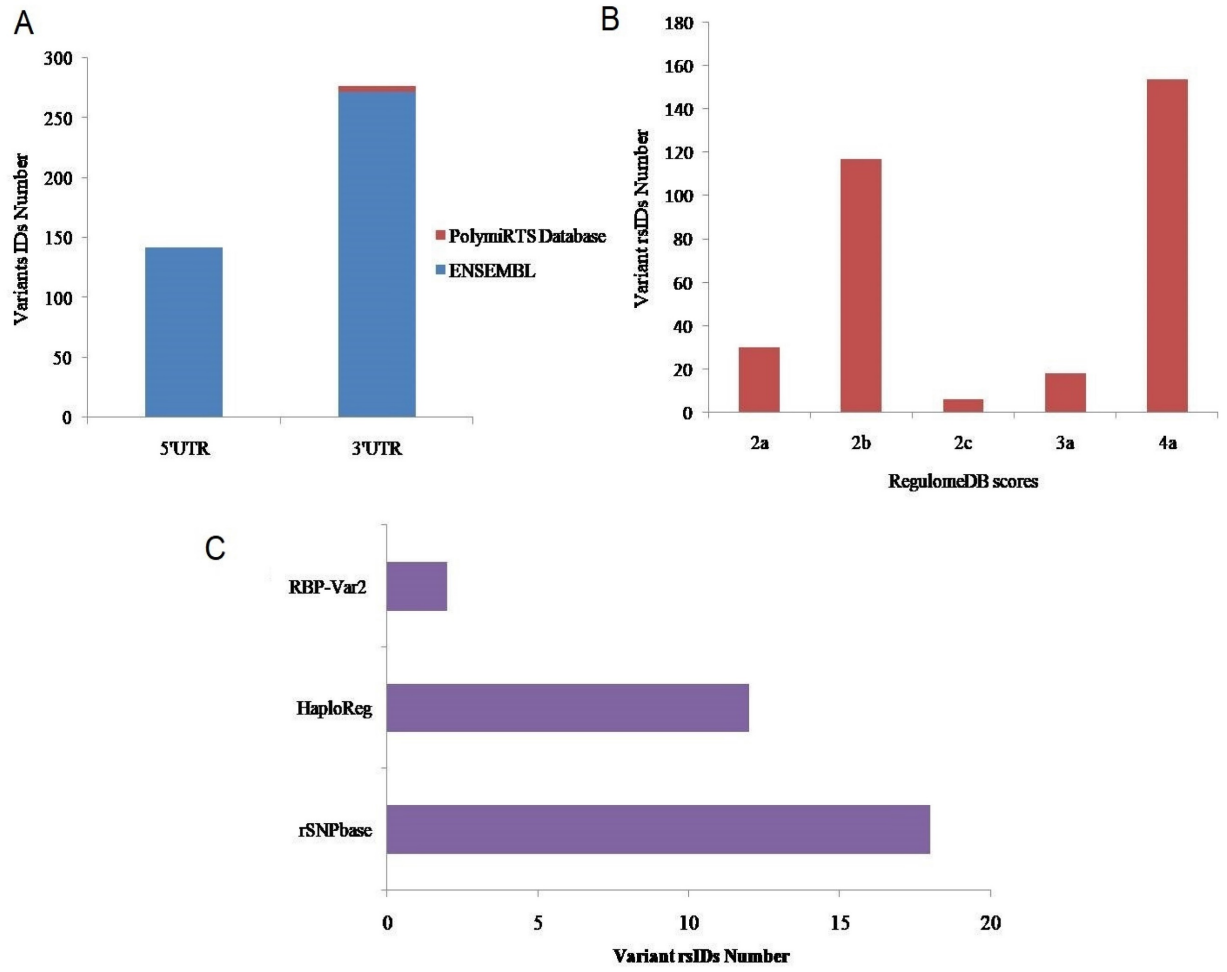
** A.** 3' and 5' UTR variants retrieved from databases. **B.** The annotated scores obtained through RegulomeDB software. **C.** Regulatory function determination of Variant rsIDs through bioinformatics.

An overview of the research study is provided in Figure 1. In total, 414 variant IDs corresponding to 272 3′ UTRs and 142 5′UTRs were retrieved from the Ensembl genome browser (Figure 2a, Supplementary File S, Table S1). Moreover, 5 IDs corresponding to 3′ UTRs were recovered from PolymiRTS Database 3.0 (Figure 2a, Supplementary Material, Table S2). From both databases, data related to genetic variants including, SNPs or SNVs was extracted while data representing indels, somatic insertion, somatic deletion, and somatic sequence alteration was filtered out. In total 419 IDs, including 414 IDs from Ensembl genome browser and 5 IDs from PolymiRTS were input into RegulomeDB software. Of the 414 IDs fetched from the Ensembl genome browser, 320 IDs have annotated scores. The annotated scores of 5 IDs from PolymiRTS Database 3.0 were also identified. The IDs corresponding to COSMIC variants were removed during the process. So, only variants provided with the valid rsIDs were included for further analysis. In total 325 rsIDs were obtained from software that had varying RegulomeDB scores from 2a to 4 (Figure 2b, Supplementary Material, Table S3 and S4). The RegulomeDB score of 2 indicated that the presence of genetic variant likely had an impact on the gene regulation process and influenced the binding of transcription factor with its target site, but as the score shifted to 3, the variant had less likely functional or regulatory role and unlikely affected binding of transcription factors with the transcription start site or DNAase hypersensitive site. Between the scores of 4-6, the experimental evidence of the genetic variant's presence to affect binding was minimal. Of 325 rsIDs, 30 rsIDs had a score of 2a, 117 rsIDs had a score of 2b, 6 rsIDs had a score of 2c, 18 rsIDs had score of 3a while 154 rsIDs had a score of 4, So, collectively, 153 rsIDs with the RegulomeDB scores between the ranges of 2a-2c likely affected binding with the transcription factors.

### Functional Annotation of 3′ and 5′ UTR Variants

**Figure 3:**
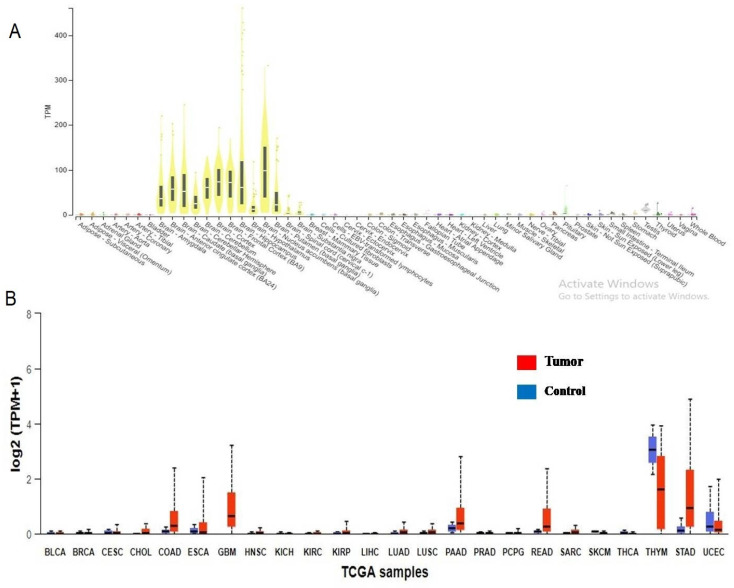
** A.** Gene expression profile of PRKCG identified in major human tissues. **B.** PRKCG expression in TCGA specific tumors in comparison with control.

The 325 annotated variant IDs retrieved from RegulomeDB software were entered into rSNPbase3.1 software and 18 variant(s) of PRKCG were involved in binding with transcription factors and regulating the CACNG7 gene (Figure 2c). From HaploReg analysis, 7 variants of PRKCG were found to localise and interact with promotor and enhancer histone markers (Figure 2c). This elaborates that these variants have potential functional roles in regulating gene expression through histone modifications in different cell types and tissues. Also, these variants were identified in the regions deemed as DNase I hypersensitive, indicating their involvement in influencing gene expression. The epigenome ID, histone Marks and DNAase hypersensitive region related to different variants have been represented as well. Also, from 325 inputs, 2 rsIDs (rs373228, rs446795) were identified using RBP-Var2 software (Figure 2c). Through CLIP technique, both the variant IDs were found in RNA regions that interact directly with RNA binding proteins. Of 2 rsIDs (rs373228, rs446795), 1 rsID (rs446795) was identified in a motif that was a riboSNitch, which indicated that because of the genetic variant, the interaction with RNA binding protein was potentially impacted, which was supported by RBP-Var2 score as well. Neither of the rsIDs were found to interact with miRNAs and the presence of these variants did not perturb interaction with miRNAs. According to Gtex software, PRKCG is expressed in multiple tissues, but its expression is high in brain cells according to RNA sequence reads number and through calculation of TPM of target gene in the studied samples (Figure 3a). When the rsIDs from PolymiRTS, rSNPbase, HaploReg and RBP-Var2 were put in Gtex software, no information related to variants was identified that needs further elucidation. To add to this, PRKCG expression across TCGA tumors indicated that it is expressed in cancers differently according to UALCAN software (Figure 3b). The Red boxplot indicated the expression of PRKCG in tumors of primary origin, while blue boxplot indicates expression in the normal samples. The high expression is indicated by the values equal to or more than 3^rd^ quartile while the values below 3^rd^ quartile indicated low or medium expression of the PRKCG.

### Impact of 3′-UTR Variants linkage with miRNA on Cancer Risk

**Figure 4:**
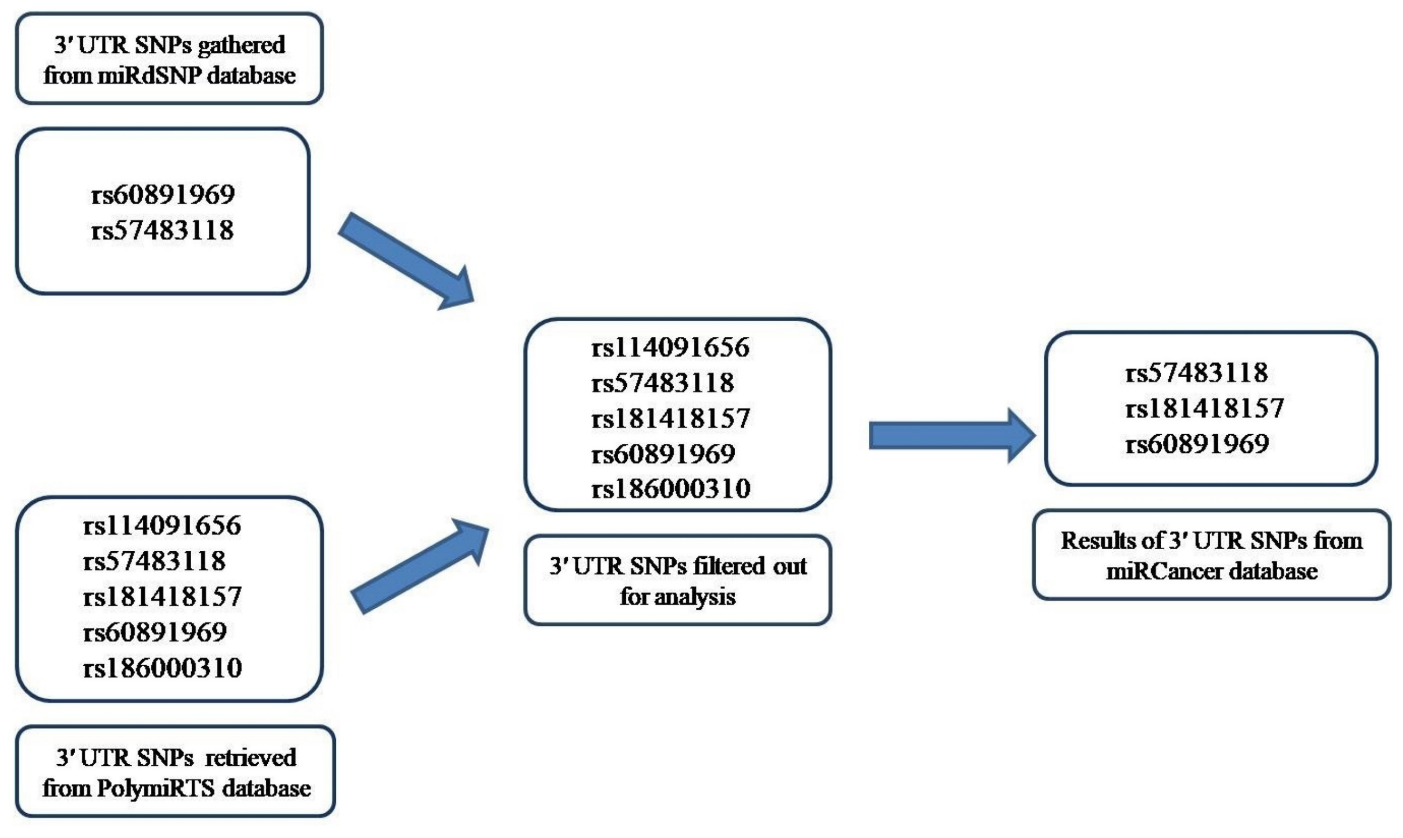
Schematic representation of gathering of data from miRdSNP and PolymiRTS databases followed by filtering step and the final result after miRCancer database analysis.

**Figure 5:**
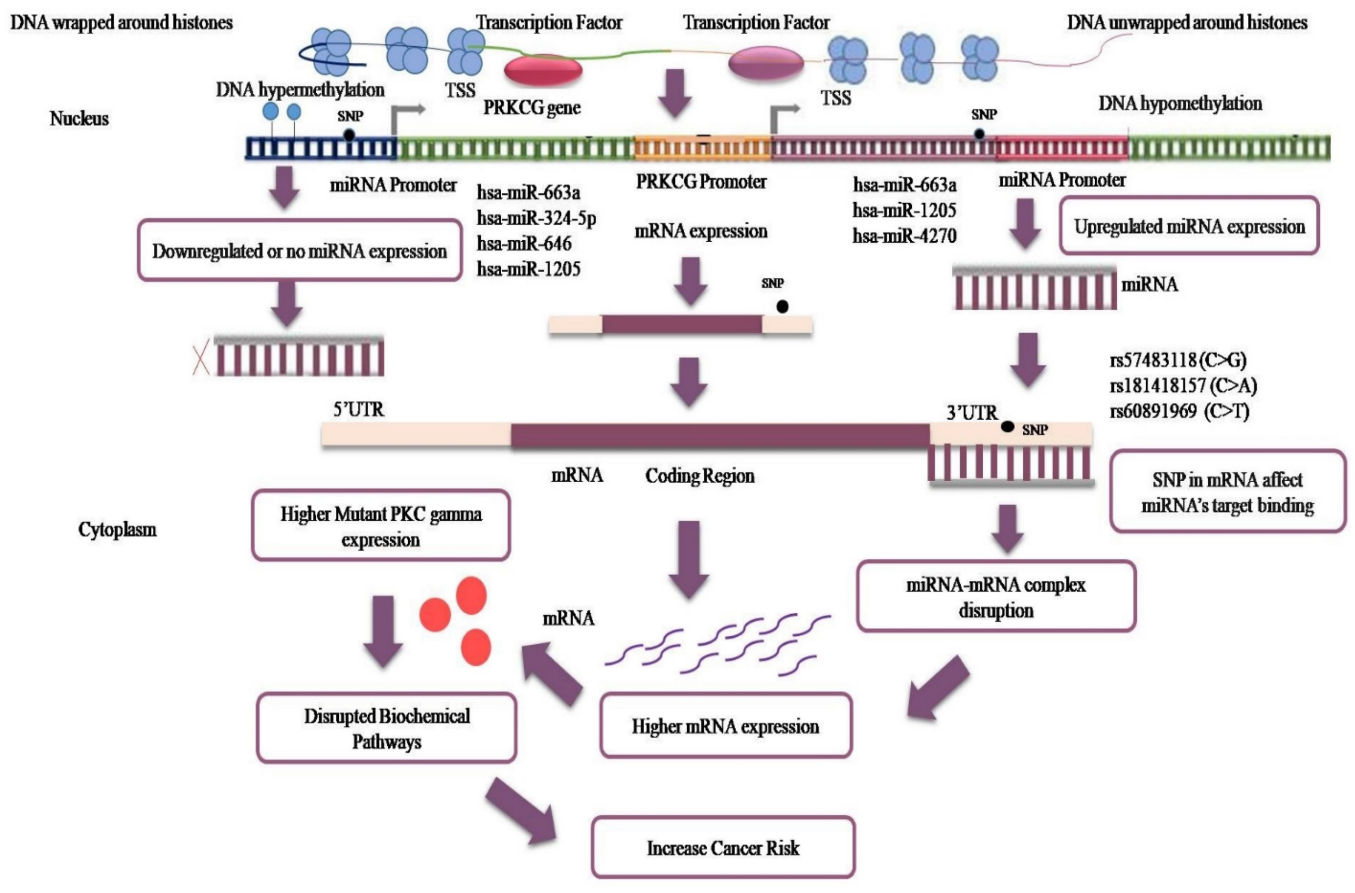
Relation between miRNA dysregulation and PRKCG over-expression in cancer cells.

**Figure 6:**
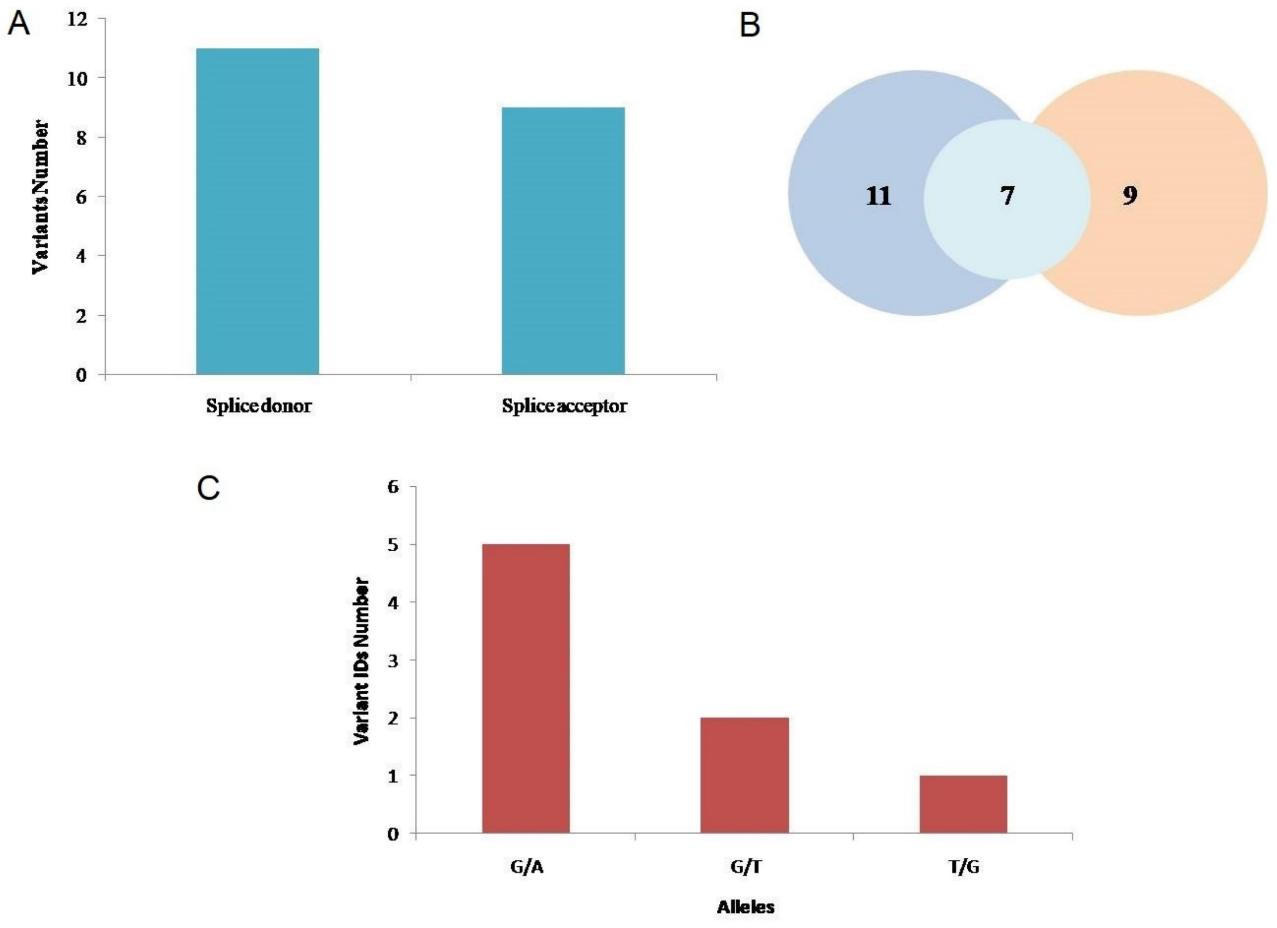
**A.** Splice donor and acceptor variants identified through ENSEMBL software. **B.** Venn diagram representing the 11 splice donor and 9 splice acceptor variants and 7 transcript rsIDs that were analyzed. **C.** Alleles and nucleotide positions (before and after mutation) of the total splice donor and splice acceptor variants.

Two 3′ UTR variants from miRdSNP database and five 3′ UTR variants from PolymiRTS database with experimentally confirmed targeting by miRNAs were analyzed. Two of the variant rsIDs were found common in both the databases and therefore, the remaining five were analyzed further. The miRNAs targeting those variants, their expression levels in different cancers were determined through miRCancer Database. The three 3′ UTR variants were separated out of five rsIDs whose targeted miRNAs had differential expression in cancers (Figure 4). The miRNAs that gave no results on miRCancer Database and whose expression were not significantly changed in cancers were not included in analysis. As noted, in most cancers, the miRNAs expression was downregulated (Figure 5). To add to this, even if the levels of the miRNAs have been upregulated but due to the SNPs presence at 3′ UTR sites, miRNAs have not been able to bind to their potential target site that has contributed to PRKCG higher expression. The miRNAs along with SNP rsIDs connected with cancer have been shown in schematic representation (Figure 6). Epigenetic aberrations that cause the wrapping or unwrapping of the histones or dysregulation in transcriptional regulatory mechanisms including hypo or hypermethylation and the presence of SNPs at the promotor regions are the primary steps that lead to less or higher expression of target genes including miRNAs. Furthermore, miRNAs including hsa-miR-663a, hsa-miR-324-5p, hsa-miR-646, hsa-miR-1205 and hsa-miR-4270 with aberrant expression in cancers have been displayed separately as downregulated or upregulated. The variants at 3′ UTR sites that act as miRNA target sites have been shown as well (Figure 6). RNAfold was used to analyze the variant rsIDs for altered RNA secondary structures by entering the miRsite sequences of target 3′ UTR variants. The effect of variants on the folded structure of RNA was found out. The lower free energy indicates stable structure of RNA. In the studied rsIDs, all the structure had negative free energy values, indicating towards the stability of structure and less potential effect of variants on RNA fold structure (Table 1).

### Splicing Variant Analysis

11 splice donor variants and 9 splice acceptor variants were identified through Ensembl genome browser software (Figure 6). After removing the 13 IDs that were COSMIC variants or were repeated and had similar chromosome positions, the remaining 7 variants IDs were further analyzed (Table 2). The MaxEntScan scores above 3.5 were interpreted to show that the respective sequences can function as the splice site. At different chromosome positions (cPos), the probability scores are given that shows the probability of authentic splice site to be functional or not with or without substitution. At 10 different chromosome positions, loss of function of authentic splice site because of variants was reported, at 1 position, gain of function while at 2 positions no effect was indicated. The likelihood that the sequence is a real splice site increases with the increasing score or positive value and decreases with the decreasing score or negative value. The higher scoring sequence has a greater chance of being used as the splice site when two sequences with different scores are shown. “No AG” or “No GT” shows the probable loss of function for authentic splice site. The SpliceAI scores of 7 rsIDs indicated that the presence of variants led to the loss of splice site's function (Table 2).

## Discussion

Gaps in knowledge continue to exist in relation to PRKCG involvement in different diseases. Past studies indicate connection of PRKCG with neurological and oncological disorders 19, 54, 55. In a notable study, its expression was found to be upregulated by 54% in colon cancer patients in comparison to normal individuals 19. Moreover, its suppression was found to reduce ability of colon cancer cells to migrate and metastasize to distant secondary sites 56. Through studies conducted *in-vivo*, immortal epithelial cells became tumorigenic with the upregulation of PRKCG 57. To add to this, PRKCG expression was reported in glioma cells 58. In another study, PRKCG transcript was observed in one of four anaplastic astrocytomas 59. In Triple-negative breast cancer (TNBC), aberrant expression of PRKCG was also noted. Moreover, lethality of inhibitor of HDAC6 was increased in TNBC because of PRKCG signaling pathway 21. PRKCG expression has not been detected in melanocytes, which requires further investigation 60. These studies indicate the importance of studying coding and non-coding SNPs of PRKCG that mainly deregulate PRKCG signaling pathways and progress the cells towards cancer. Past studies highlight the prominent role of non-synonymous (ns) SNPs in carcinogenesis. These nsSNPs form the part of coding region of PRKCG. In a study, the pathogenic non-synonymous SNP rs386134171 of PRKCG was found to destabilize the structure of PRKCG and alter its function that leads to HCC 23. In another study, positive association of PRKCG non-synonymous variant rs1331262028 with ovarian cancer was established 22.

Furthermore, the presence of SNP rs454006 in PRKCG was linked with increased risk of osteosarcoma 55. This SNP was identified in the non-coding region i.e. intron 3 region of PRKCG 18. Previously, no study has demonstrated the role of non-coding variants that reside in 5′UTR, 3′UTR, splice donor and acceptor sites with cancer susceptibility. The current study has utilized *in-silico* approaches to understand the PRKCG gene and the effects of its non-coding variants on post-transcriptional and translational mechanisms. The regulatory roles of 5′ and 3′UTR variants were evaluated using RegulomeDB 29, rSNPbase 61, HaploReg 62, and RBP-Var2 63.

The RegulomeDB software incorporates data from experimental techniques and from ENCODE project to gain knowledge regarding gene regulation mechanisms and variant IDs that are entered into the software 64. The scoring system provided by the software helps in the elucidation of functional components including TF interaction, chromatin structure and histone modification. One of the important conclusions from the ENCODE research is that there is a complicated interaction between TFs and other genome components because of chromatin shape and histone modification. Different forms of histone modifications have been demonstrated to be related to active or repressed chromatin states, which in turn might alter the binding of TFs to the surrounding DNA sequences. Similarly, the spatial structure of DNA inside the nucleus may also influence gene expression, since TFs may be more or less accessible to their target sites depending on their placement within the genome. Moreover, there is an increasing amount of data to imply that TFs play a crucial role in gene regulation. For example, high-throughput functional assays such as ChIP-seq and DNase I-hypersensitive site sequencing experimentally aid in discovering functional areas for TF binding 65.

Another major area of study in the realm of gene regulation is the function of genetic variation in modifying TF binding. It has been demonstrated that variants in genes may lead to variations in TF binding, which can in turn impact gene expression and contribute to disease risk. In one study that was directed towards FGFR2, it was noted that because of the single nucleotide changes in the sequence of Intron 2 at two positions, the reported altered binding of transcription factors lead to increase expression of FGFR2 in breast cancer 66. Thus, variants cause both up-and down-regulation of gene expression 67. rSNPbase3.1 and HaploReg software 32 provided additional information on variant-related post-transcriptional regulatory activity in the study. HaploReg identified promotor and enhancer histone marks and DNAase hypersensitivity of variants. Further, the RBP-Var2 database identified variants that affects post-transcriptional mechanisms by altering RNA binding patterns 34.

The relationship between 3′ and 5′ UTR variants and gene expression across multiple human tissues was determined utilizing Gtex software 68. With the help of Gtex software 69, we ascertained the PRKCG and the variants expression in human tissues. Through UALCAN 37, gene expression was identified in TCGA cancers. In addition to 5′UTR and 3′UTR variants, our study focused on splice donor and acceptor sites. These sites are positioned at the intersections of exons and introns in a pre-mRNA molecule. The splice donor site is positioned at the 5' end of an intron and is identified by the splicing machinery that recognizes the splice donor site through attaching with the GU conserved nucleotide sequence in humans. On the other hand, the splice acceptor site is identified near the 3' end of an intron and is also recognized by the same splicing machinery through the AG conserved sequence of nucleotides. The splicing mechanisms splice out the introns for the functional protein generation 70. Notably, variants at these junctions can perturb the splicing mechanisms that lead to altered protein sequence and structures, contributing to disease pathology 70.

Moreover, splice site consensus sequences present at exon-intron junctions are phylogenetically conserved. Understanding splicing mechanisms requires the recognition of splice site motifs 71. Nine splice acceptor variations and eleven splice donor variations were studied to see if they had a significant impact on splice sites. The entropy model-based software, MaxEntScan, was used to estimate whether a specific sequence motif would act as a donor or acceptor splice site. The variations on splice sites could lead to formation of a new splice site or deletion of the authentic one that can vary withMaxENT scores. For example, a normal splice site might have a MaxENTscore of 7.84 while the mutated splice site has a MaxENT score of 10.42 72. Along with MaxENTScan, the scores of SpliceAI 73 also provided thorough and detailed information regarding the functionality of spice sites.

Further, in this study, we have taken into consideration the involvement of miRNAs in causing perturbed gene expression due to alteration in miRNA target sites on the mRNA. miRNAs are intriguing molecular players for gene regulation and are associated with numerous human disorders. Importantly, variants found in the target sequences of these miRNAs lead to dysregulated gene expression which may cause disease advancement 58. Moreover, gene expressions that encode miRNAs get repressed or down regulated, one reason of which is the presence of variants in the promoter. The resultant gene, in this case miRNA, is not expressed that leads to increased expression of the targeted gene 36, 59. Furthermore, miRNAs can act as oncogenes or as tumor suppressors and their aberrant expression levels contribute to oncogenesis 37.

Moreover, because of 3'UTR variants, the interaction of mRNA with miRNA gets disrupted that also causes the expression of targeted protein to increase that finally leads to higher cancer risk when biochemical pathways are disrupted 37, 60, 61. Moreover, the miRNA expression upregulation and downregulation get influenced by epigenetic changes that lead to changes on the histone's marks and cause DNA unwrapping or wrapping around histones 34. To add to this, hypo and hypermethylation and the presence of variants in the promotor also impact expression of genes that encode miRNAs 35, 36.

In the current study, we analyze genetic variants in the miRNA sites. Not only are the variants in the target sites of miRNA functionally important but also the variants that originate in the miRNAs. In a previous study, miR-146a polymorphism, rs2910164, including a G>C nucleotide variation on the seed region of miR146a-3p caused the conversion of G:U pair to a C:U. This mismatch resulted in changing the selectivity of mature miR-146a binding to its targets that contributed to higher production of miR-146a in cancers 74.

Our study presently focused on studying 3′UTRs in relation to interaction with miRNAs. Although miRNAs may target 5′UTRs, their impact when compared to 3′UTR regions is limited 40. In one study, variant rsIDs including rs12516, rs3092995 and rs8176318 that were noted in 3′UTR of BRCA1 affected the interaction with the miR-103 seed sequence that caused the breast cancer risk to increase in the study group of African American women 75. miRdSNP and PolymiRTS databases 76 were explored for identification of miRNAs that target 3′UTR sites. For the miRNAs that were found in the study to bind with variants at 3′UTR, their expression levels were assessed in different cancers through the miRCancer Database 44. Moreover, when the conserved or non-conserved site is disrupted, it impacts miRNA targeting. Genetic variants significantly affect miRNA target sites, which is determined through scores 42. In our study, we used RNAfold 77 to determine the altered secondary structure of 3′UTR variants whose binding with miRNAs gets disrupted because of genetic variants.

This study has covered *in-silico* analysis to explore the potential mechanisms affected at molecular level because of non-coding variants. The *in-silico* approach provided a cost and time-efficient means of screening a vast array of non-coding variants for further experimental validation. The wet lab experiments that are lacking in the current study would be part of future studies. Moreover, for the outcomes of the current study to have significant clinical implications and applications, this study would be conducted in a larger cohort. Our future explorations involve the validation of obtained *in-silico* results through *in-vitro* and *in-vivo* methods. Further investigations would take into consideration the use of surgically resected cancer tissue and cancer cell line for reliable results. The non-coding variants can interfere with the transcription factors binding and affect normal function of gene regulatory elements. Moreover, the presence of SNPs in the enhancer and silencer that form the part of non-coding genomic regions 78, also affect the regulatory mechanisms that lead to cancer 79. This study would be utilized to study non-coding SNPs, specifically in relation to enhancer and silencer elements. Notably, the 3'UTR, 5' UTRs and splice sites would be studied through deep sequencing technology. Past studies have indicated different molecular pathways of PRKCG with reference to cancer 19, 23. This study would be utilized to unveil the role of non-coding SNPs in impacting PRKCG signalling pathways. A past study compares the expression of non-phosphorylated and Thr^514^-phosphorylated form of PRKCG in colon cancer cells 80. So, PTMs can alter the expression of PRKCG in cancer cells. Our further investigations would include the identification of PTMs of PRKCG that could affect transcription and translation processes.

## Conclusion

This study analyzed the non-coding variants of the PRKCG gene, especially present within the 3'UTR, 5' UTR, splice donor and acceptor sites. The presence of pathogenic non-coding SNPs affected the binding of transcription factors with the regulatory elements. Moreover, the mRNA interactions with miRNAs were also impacted because of 3'UTR variants. The splice site variants caused the loss of donor and acceptor sites that influenced the splicing mechanisms. The study also covered the effect of presence of non-coding variants in DNase hypersensitive regions. Also, the effect of change in histone modifications and chromatin architecture on transcription mechanisms was briefly studied.

## Supplementary Material

Supplementary tables.

## Figures and Tables

**Table 1:** Variant rsIDs with their changed secondary structures identified through RNAfold

sIDs	Target site (miRsite) sequences	Free energy (kcal/mol)	Frequency of MFE (Minimum Free Energy) structure	Graphical output (MFE secondary structure)
rs57483118	GaggctGCCCGCC	-2.63	95.50%	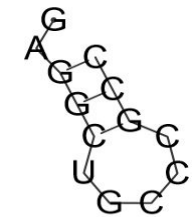
	GggATGGTGATG	-0.02	97.33 %	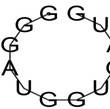
rs181418157	AcgtccAGCTGCT	-0.10	85.36%	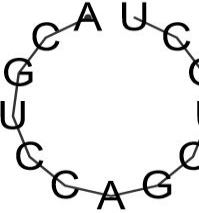
rs60891969	TctcCCTGCAGcc	-0.01	98.58%	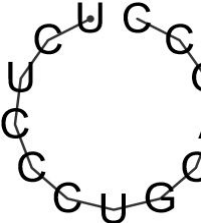
	TCTCCCTGcagcc	-0.01	98.58%	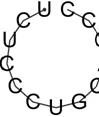
	GggATGTTGATG	-0.02	97.02 %	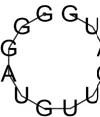

**Table 2:** Interpretation by MaxEntScan software

rsIDs	cPos	Score	Score Interpretation (Effect on authentic splice site)	MaxEntScan scores
rs1344692203	1: 285+1	+45.59% (reference)	Loss of Function	9.22
		<no GT> (variant)		
rs1568755566	1: 822-2	+5.96% (reference)	Loss of Function	7.09
		<no AG> (variant)		
	2: 822:1	<no AG> (reference)	Loss of Function	
		-33.56% (variant)		2.07
rs1395398748	1: 909+1	+20.00% (reference)	No predictive effect	7.84
		+20.00% (variant)		7.84
	2: 939	<no GT> (reference)	Loss of Function	-23.68
		-89.54% (variant)		
	3: 939+1	+82.62% (reference)	Loss of Function	10.03
		<no GT> (variant)		
rs1384774676	1:1282-2	+32.08% (reference)	Loss of Function	9.07
		<no AG> (variant)		
	2:1282-1	<no AG> (reference)	Gain of Function	
		+0.49% (variant)		0.56
rs59309543	1:1576-2	+55.16% (reference)	Loss of Function	10.14
		<no AG> (variant)		
rs1599953647	1:1656+1	+87.92% (reference)	Loss of Function	11.08
		<no GT> (variant)		
	2:1656+11	+42.96% (reference)	No predictive effect	5.87
		+42.96% (variant)		5.87
rs1406338491	1:1764	<no GT> (reference)	Loss of Function	
		-67.95% (variant)		-11
	2:1764+1	+26.51% (reference)	Loss of Function	4.74
		<no GT> (variant)		
