# Supplementary material for: Genetic Variants at PRKCG Splice and UTR Sites Promote Cancer Susceptibility by Disrupting Epigenetic and miRNA Regulatory Network
Source: J Cancer. 2024 Oct 28;15(20):6644–57. doi: 10.7150/jca.100911 (PMC11632988; doi:10.7150/jca.100911)
Supplement: Supplementary file 1 — Supplementary tables. [file jcav15p6644s1.pdf]

Supplementary file S1

Genetic Variants at PRKCG Splice and UTR Sites Promote Cancer Susceptibility by Disrupting Epigenetic and miRNA Regulatory Network

Fizzah Abid[1], Khushbukhat Khan[1], Naeem Mahmood Ashraf[2], Yasmin Badshah[1], Maria Shabbir[2], Janeen H, Trembley[,3,4,5], Tayyaba Afsar[6], Ali Almajwal[6], Suhail Razak[6*].

TableS1

Variant ID	Chr: bp	vf_allele
rs976073603	19:53882199	G
rs1276051930	19:53882204	G
rs1436601889	19:53882208	C
rs1052659074	19:53882209	A
rs1393619899	19:53882210	C
rs1441959659	19:53882211	T
rs892298039	19:53882213	G
COSV54730760	19:53882224	COSMIC_MUTATION
rs923521166	19:53882226	T
rs1296005369	19:53882227	T
rs949277052	19:53882228	T
rs750821949	19:53882231	T
rs909407651	19:53882235	A
rs942158836	19:53882236	T
rs1192949516	19:53882238	T
rs1478418119	19:53882241	T
rs769506025	19:53882242	A
rs1262712418	19:53882244	A
rs1325591802	19:53882246	T
rs905131949	19:53882249	T
rs1350550050	19:53882252	A
rs1489148254	19:53882256	G
rs1286385136	19:53882257	T
rs761232820	19:53882259	T
rs766844154	19:53882260	A
rs752173466	19:53882262	T
rs1278778508	19:53882263	A
rs1278778508	19:53882263	G
rs757854018	19:53882266	A
rs757854018	19:53882266	C
rs763607343	19:53882272	A
rs763607343	19:53882272	C
rs373228	19:53882273	C
rs446795	19:53882274	G
rs756925547	19:53882276	T
rs1289889326	19:53882281	A
rs1242794867	19:53882282	T
rs781024396	19:53882283	T
rs745696394	19:53882284	T
rs1599936643	19:53882286	C

rs756043883	19:53882287	T
rs780097009	19:53882289	A
rs780097009	19:53882289	T
rs749263686	19:53882290	T
rs1363639422	19:53882293	T
rs1468742683	19:53882294	T
COSV54725002	19:53882296	COSMIC_MUTATION
rs1414571673	19:53882297	T
rs768803096	19:53882298	T
rs774579501	19:53882300	A
rs774579501	19:53882300	G
rs1599936693	19:53882301	A
COSV99669472	19:53882301	COSMIC_MUTATION
rs748380013	19:53882305	A
rs748380013	19:53882305	G
COSV54726813	19:53882305	COSMIC_MUTATION
rs772242661	19:53882312	C
rs773605647	19:53882314	T
rs1246484792	19:53882316	T
rs760985936	19:53882317	T
rs766798511	19:53882318	A
rs1249568890	19:53882322	C
rs1230895829	19:53882323	T
rs1259510289	19:53882324	T
rs1178925213	19:53882325	G
rs777064041	19:53882326	A
rs777064041	19:53882326	C
rs954889760	19:53882329	T
rs760040558	19:53882334	T
rs763588729	19:53882336	T
rs1478893732	19:53882337	A
rs1478893732	19:53882337	T
rs1181029345	19:53882338	A
rs994248167	19:53882339	A
rs751126244	19:53882340	C
rs1189819095	19:53882342	A
rs756908303	19:53882345	A
rs1210814979	19:53882349	A
rs1475025188	19:53882352	C
rs767113684	19:53882355	G
COSV54729700	19:53882357	COSMIC_MUTATION
rs750117911	19:53882359	A

rs750117911	19:53882359	T
rs780012764	19:53882360	A
COSV99668990	19:53882360	COSMIC_MUTATION
rs988069778	19:53882361	C
COSV54734572	19:53882361	COSMIC_MUTATION
rs574559869	19:53882362	T
rs755036563	19:53882363	T
rs1334621754	19:53882368	T
COSV99668358	19:53882368	COSMIC_MUTATION
rs1318118581	19:53882369	T
rs1354427343	19:53882370	T
rs779124246	19:53882371	G
rs779124246	19:53882371	T
rs1395783139	19:53882372	C
COSV54732369	19:53882373	COSMIC_MUTATION
rs748227994	19:53882375	A
rs1188066946	19:53882376	T
rs1305112576	19:53882378	C
rs772306670	19:53882379	T
rs535615037	19:53882380	T
rs1421758670	19:53882386	T
COSV54730211	19:53882386	COSMIC_MUTATION
rs1034219389	19:53882387	A
rs1251263271	19:53882391	C
COSV54724663	19:53882394	COSMIC_MUTATION
rs773267630	19:53882396	T
rs553905626	19:53882397	C
rs553905626	19:53882397	G
rs771133027	19:53882399	A
rs771133027	19:53882399	C
rs1485459135	19:53882401	T
rs777045504	19:53882402	G
rs760022048	19:53882405	T
rs1429222917	19:53882406	C
rs765779813	19:53882407	G
COSV99669381	19:53882417	COSMIC_MUTATION
rs1164408511	19:53882421	T
COSV99669516	19:53882428	COSMIC_MUTATION
COSV99668845	19:53882435	COSMIC_MUTATION
rs1337423686	19:53882436	T
COSV99668361	19:53882438	COSMIC_MUTATION
rs773816756	19:53882449	T

Table S2

Chr:bp	dbSNP ID	Variant type	Wobble base pair
19:54410348	rs114091656	SNP	N
19:54410577	rs57483118	SNP	N
19:54410637	rs181418157	SNP	N
19:54410745	rs60891969	SNP	N
19:54410819	rs186000310	SNP	N

TableS3

Chromosome location	dbSNP IDs	Rank
chr19:54385452..54385453	rs976073603	4
chr19:54385457..54385458	rs1276051930	2b
chr19:54385461..54385462	rs1436601889	2b
chr19:54385462..54385463	rs1052659074	2b
chr19:54385463..54385464	rs1393619899	2b
chr19:54385464..54385465	rs1441959659	2b
chr19:54385466..54385467	rs892298039	2b
chr19:54385479..54385480	rs923521166	2b
chr19:54385480..54385481	rs1296005369	4
chr19:54385481..54385482	rs949277052	4
chr19:54385484..54385485	rs750821949	2a
chr19:54385488..54385489	rs909407651	2a
chr19:54385489..54385490	rs942158836	2a
chr19:54385491..54385492	rs1192949516	2a
chr19:54385494..54385495	rs1478418119	4
chr19:54385495..54385496	rs769506025	4
chr19:54385497..54385498	rs1262712418	2b
chr19:54385499..54385500	rs1325591802	2b
chr19:54385502..54385503	rs905131949	2b
chr19:54385505..54385506	rs1350550050	2b
chr19:54385509..54385510	rs1489148254	2b
chr19:54385510..54385511	rs1286385136	2b
chr19:54385512..54385513	rs761232820	2b
chr19:54385513..54385514	rs766844154	4
chr19:54385515..54385516	rs752173466	4
chr19:54385516..54385517	rs1278778508	4
chr19:54385519..54385520	rs757854018	4
chr19:54385525..54385526	rs763607343	4
chr19:54385526..54385527	rs373228	4
chr19:54385527..54385528	rs446795	4
chr19:54385529..54385530	rs756925547	4
chr19:54385534..54385535	rs1289889326	2b
chr19:54385535..54385536	rs1242794867	2b
chr19:54385536..54385537	rs781024396	2b
chr19:54385537..54385538	rs745696394	2b
chr19:54385540..54385541	rs756043883	2b
chr19:54385542..54385543	rs780097009	4
chr19:54385543..54385544	rs749263686	4
chr19:54385546..54385547	rs1363639422	2a
chr19:54385547..54385548	rs1468742683	2a

chr19:54385550..54385551	rs1414571673	2a
chr19:54385551..54385552	rs768803096	2a
chr19:54385553..54385554	rs774579501	2a
chr19:54385558..54385559	rs748380013	2a
chr19:54385565..54385566	rs772242661	4
chr19:54385567..54385568	rs773605647	4
chr19:54385569..54385570	rs1246484792	4
chr19:54385570..54385571	rs760985936	4
chr19:54385571..54385572	rs766798511	4
chr19:54385575..54385576	rs1249568890	4
chr19:54385576..54385577	rs1230895829	4
chr19:54385577..54385578	rs1259510289	4
chr19:54385578..54385579	rs1178925213	4
chr19:54385579..54385580	rs777064041	4
chr19:54385582..54385583	rs954889760	4
chr19:54385587..54385588	rs760040558	4
chr19:54385589..54385590	rs763588729	4
chr19:54385590..54385591	rs1478893732	4
chr19:54385591..54385592	rs1181029345	4
chr19:54385592..54385593	rs994248167	4
chr19:54385593..54385594	rs751126244	4
chr19:54385595..54385596	rs1189819095	4
chr19:54385598..54385599	rs756908303	2b
chr19:54385602..54385603	rs1210814979	2b
chr19:54385605..54385606	rs1475025188	2b
chr19:54385608..54385609	rs767113684	2b
chr19:54385612..54385613	rs750117911	2b
chr19:54385613..54385614	rs780012764	3a
chr19:54385614..54385615	rs988069778	3a
chr19:54385615..54385616	rs574559869	4
chr19:54385616..54385617	rs755036563	4
chr19:54385621..54385622	rs1334621754	2b
chr19:54385622..54385623	rs1318118581	2b
chr19:54385623..54385624	rs1354427343	2b
chr19:54385624..54385625	rs779124246	2b
chr19:54385625..54385626	rs1395783139	2b
chr19:54385628..54385629	rs748227994	2b
chr19:54385629..54385630	rs1188066946	2b
chr19:54385631..54385632	rs1305112576	2b
chr19:54385632..54385633	rs772306670	2b
chr19:54385633..54385634	rs535615037	2b
chr19:54385639..54385640	rs1421758670	2b

TableS 4: RegulomeDB software scores of 3′ and 5′ UTR variants

Scores	3 prime UTR variant	5 prime UTR variant	Grand Total
**4**	106	48	154
**2a**	20	10	30
**2b**	72	45	117
**2c**	4	2	6
**3a**	16	2	18
**Grand Total**	**218**	**107**	**325**

TableS 5: SNP rsIDs corresponding to 3’ and 5’ prime UTRs with transcription factor binding role, identified through rSNPbase3.1.

rs_IDs	Chromosome	Allele	Related regulatory elements	Target genes
rs778703745	chr19:54410699	C/T	TF binding region	CACNG7
rs938508106	chr19:54410715	C/T	TF binding region	CACNG7
rs1057182500	chr19:54410716	A/T	TF binding region	CACNG7
rs60891969	chr19:54410745	C/T	TF binding region	CACNG7
rs999473059	chr19:54410759	C/G	TF binding region	CACNG7
rs1031848033	chr19:54410763	C/T	TF binding region	CACNG7
rs753142841	chr19:54410764	A/G	TF binding region	CACNG7
rs1009068182	chr19:54410781	C/T	TF binding region	CACNG7
rs565287029	chr19:54410785	C/T	TF binding region	CACNG7
rs1001200260	chr19:54410788	A/C	TF binding region	CACNG7
rs1034552653	chr19:54410798	C/T	TF binding region	CACNG7
rs368537194	chr19:54410799	C/T	TF binding region	CACNG7
rs186000310	chr19:54410819	C/T	TF binding region	CACNG7
rs975121038	chr19:54410835	C/T	TF binding region	CACNG7

TableS 6: Functional Analysis of 3’ and 5’ UTR SNPs using HaploReg v4.1

rsID	Chr:Position	GENCODE genes	dbSNP function Annotation	Ref/Alt	Promotor Histone Marks	Enhancer Histone Marks	Motifs Changed	DNaseHypersentivity
rs373228	19: 53882273	PRKCG	5' UTR	G/C	17 tissues	15 tissues	7 altered motifs	34 tissues
rs446795	19:53882274	PRKCG	5' UTR	C/G	17 tissues	15 tissues	4 altered motifs	34 tissues
rs114091656	19:53907094	PRKCG	3' UTR	C/T	5 tissues	12 tissues		14 tissues
rs57483118	19:53907323	PRKCG	3' UTR	C/G	7 tissues	11 tissues	8 altered motifs	7 Tissues
rs181418157	19:53907383	PRKCG	3' UTR	C/A	6 tissues	10 tissues	7 altered motifs	5 tissues
rs60891969	19:53907491	PRKCG	3' UTR	C/A	6 tissues	10 tissues	8 altered motifs	5 tissues
rs186000310	19:53907565	PRKCG	3' UTR	T/C	6 tissues	11 tissues	4 altered motifs	5 tissues

TableS7

**Epigenome ID (EID)**	**Group**	**Mnemonic**	**Description**
E017	IMR90	LNG.IMR90	IMR90 fetal lung fibroblasts Cell Line
E002	ESC	ESC.WA7	ES-WA7 Cells
E008	ESC	ESC.H9	H9 Cells
E001	ESC	ESC.I3	ES-I3 Cells
E015	ESC	ESC.HUES6	HUES6 Cells
E014	ESC	ESC.HUES48	HUES48 Cells
E016	ESC	ESC.HUES64	HUES64 Cells
E003	ESC	ESC.H1	H1 Cells
E024	ESC	ESC.4STAR	ES-UCSF4 Cells
E020	iPSC	IPSC.20B	iPS-20b Cells
E019	iPSC	IPSC.18	iPS-18 Cells
E018	iPSC	IPSC.15b	iPS-15b Cells
E021	iPSC	IPSC.DF.6.9	iPS DF 6.9 Cells
E022	iPSC	IPSC.DF.19.11	iPS DF 19.11 Cells
E007	ES-deriv	ESDR.H1.NEU	H1 Derived Neuronal Progenitor Cultured Cel
E009	ES-deriv	ESDR.H9.NEU	H9 Derived Neuronal Progenitor Cultured Cel
E010	ES-deriv	ESDR.H9.NEU	H9 Derived Neuron Cultured Cells
E013	ES-deriv	ESDR.CD56.M	hESC Derived CD56+ Mesoderm Cultured Ce
E012	ES-deriv	ESDR.CD56.E	hESC Derived CD56+ Ectoderm Cultured Cel
E011	ES-deriv	ESDR.CD184.	hESC Derived CD184+ Endoderm Cultured C
E004	ES-deriv	ESDR.H1.BMI	H1 BMP4 Derived Mesendoderm Cultured Ce
E005	ES-deriv	ESDR.H1.BMI	H1 BMP4 Derived Trophoblast Cultured Cells
E006	ES-deriv	ESDR.H1.MS(	H1 Derived Mesenchymal Stem Cells
E062	Blood & T-cell	BLD.PER.MO	Primary mononuclear cells from peripheral blo
E034	Blood & T-cell	BLD.CD3.PPC	Primary T cells from peripheral blood
E045	Blood & T-cell	BLD.CD4.CD2	Primary T cells effector/memory enriched fron
E033	Blood & T-cell	BLD.CD3.CPC	Primary T cells from cord blood
E044	Blood & T-cell	BLD.CD4.CD2	Primary T regulatory cells from peripheral blo
E043	Blood & T-cell	BLD.CD4.CD2	Primary T helper cells from peripheral blood
E039	Blood & T-cell	BLD.CD4.CD2	Primary T helper naive cells from peripheral b
E041	Blood & T-cell	BLD.CD4.CD2	Primary T helper cells PMA-I stimulated
E042	Blood & T-cell	BLD.CD4.CD2	Primary T helper 17 cells PMA-I stimulated
E040	Blood & T-cell	BLD.CD4.CD2	Primary T helper memory cells from periphera
E037	Blood & T-cell	BLD.CD4.MP(	Primary T helper memory cells from periphera
E048	Blood & T-cell	BLD.CD8.MP(	Primary T CD8+ memory cells from periphera

E038	Blood & T-cell	BLD.CD4.NPC	Primary T helper naive cells from peripheral b
E047	Blood & T-cell	BLD.CD8.NPC	Primary T CD8+ naive cells from peripheral b
E029	HSC & B-cell	BLD.CD14.PC	Primary monocytes from peripheral blood
E031	HSC & B-cell	BLD.CD19.CP	Primary B cells from cord blood
E035	HSC & B-cell	BLD.CD34.PC	Primary hematopoietic stem cells
E051	HSC & B-cell	BLD.MOB.CD	Primary hematopoietic stem cells G-CSF-mob
E050	HSC & B-cell	BLD.MOB.CD	Primary hematopoietic stem cells G-CSF-mob
E036	HSC & B-cell	BLD.CD34.CC	Primary hematopoietic stem cells short term cu
E032	HSC & B-cell	BLD.CD19.PP	Primary B cells from peripheral blood
E046	HSC & B-cell	BLD.CD56.PC	Primary Natural Killer cells from peripheral bl
E030	HSC & B-cell	BLD.CD15.PC	Primary neutrophils from peripheral blood
E026	Mesench	STRM.MRW.M	Bone Marrow Derived Cultured Mesenchymal
E049	Mesench	STRM.CHON.	Mesenchymal Stem Cell Derived Chondrocyte
E025	Mesench	FAT.ADIP.DR	Adipose Derived Mesenchymal Stem Cell Cul
E023	Mesench	FAT.MSC.DR.	Mesenchymal Stem Cell Derived Adipocyte C
E052	Myosat	MUS.SAT	Muscle Satellite Cultured Cells
E055	Epithelial	SKIN.PEN.FRS	Foreskin Fibroblast Primary Cells skin01
E056	Epithelial	SKIN.PEN.FRS	Foreskin Fibroblast Primary Cells skin02
E059	Epithelial	SKIN.PEN.FRS	Foreskin Melanocyte Primary Cells skin01
E061	Epithelial	SKIN.PEN.FRS	Foreskin Melanocyte Primary Cells skin03
E057	Epithelial	SKIN.PEN.FRS	Foreskin Keratinocyte Primary Cells skin02
E058	Epithelial	SKIN.PEN.FRS	Foreskin Keratinocyte Primary Cells skin03
E028	Epithelial	BRST.HMEC.3	Breast variant Human Mammary Epithelial Ce
E027	Epithelial	BRST.MYO	Breast Myoepithelial Primary Cells
E054	Neurosph	BRN.GANGEM	Ganglion Eminence derived primary cultured r
E053	Neurosph	BRN.CRTX.D	Cortex derived primary cultured neurospheres
E112	Thymus	THYM	Thymus
E093	Thymus	THYM.FET	Fetal Thymus
E071	Brain	BRN.HIPP.MI	Brain Hippocampus Middle
E074	Brain	BRN.SUB.NIC	Brain Substantia Nigra
E068	Brain	BRN.ANT.CA	Brain Anterior Caudate
E069	Brain	BRN.CING.GY	Brain Cingulate Gyrus
E072	Brain	BRN.INF.TMP	Brain Inferior Temporal Lobe
E067	Brain	BRN.ANG.GY	Brain Angular Gyrus
E073	Brain	BRN.DL.PRFF	Brain_Dorsolateral_Prefrontal_Cortex
E070	Brain	BRN.GRM.MT	Brain Germinal Matrix
E082	Brain	BRN.FET.F	Fetal Brain Female
E081	Brain	BRN.FET.M	Fetal Brain Male
E063	Adipose	FAT.ADIP.NU	Adipose Nuclei
E100	Muscle	MUS.PSOAS	Psoas Muscle
E108	Muscle	MUS.SKLT.F	Skeletal Muscle Female
E107	Muscle	MUS.SKLT.M	Skeletal Muscle Male
E089	Muscle	MUS.TRNK.F	Fetal Muscle Trunk
E090	Muscle	MUS.LEG.FET	Fetal Muscle Leg

TableS 8: rsIDs identified through RBP-Var2 software

Chr:Position	rsIDs	CLIP binding	Motif matching	riboSNitch	eQTL	miRNA targets	RBP-Var score
19:53882273	rs373228	Yes	No	No	No	No	6
19:53882274	rs446795	Yes	Yes	Yes	No	No	2c

Tab leS9

rs1027045026	chr19:54410846	C/T	TF binding region	CACNG7
rs952664909	chr19:54410849	C/T	TF binding region	CACNG7
rs756382773	chr19:54410864	A/G	TF binding region	CACNG7
rs544511515	chr19:54410900	C/T	TF binding region	CACNG7

TableS10

Variant ID	Chr: bp	vf_allele	Alleles
COSV54730300	19:53883162	COSMIC_MUTATION	COSMIC_MUTATION
rs1344692203	19:53884244	A	G/A
COSV105040002	19:53884245	COSMIC_MUTATION	COSMIC_MUTATION
rs1395398748	19:53893392	T	G/T
COSV54731417	19:53900325	COSMIC_MUTATION	COSMIC_MUTATION
COSV99669045	19:53900483	COSMIC_MUTATION	COSMIC_MUTATION
COSV54724175	19:53900750	COSMIC_MUTATION	COSMIC_MUTATION
rs1599953647	19:53903155	G	T/G
rs1406338491	19:53904743	A	G/A/T
rs1406338491	19:53904743	T	G/A/T
COSV54734075	19:53904743	COSMIC_MUTATION	COSMIC_MUTATION
COSV54735488	19:53904743	COSMIC_MUTATION	COSMIC_MUTATION
rs1568755566	19:53892987	A	G/A
COSV54730811	19:53892987	COSMIC_MUTATION	COSMIC_MUTATION
COSV54730078	19:53898438	COSMIC_MUTATION	COSMIC_MUTATION
COSV99669560	19:53898438	COSMIC_MUTATION	COSMIC_MUTATION
rs1384774676	19:53900232	A	G/A
COSV54732533	19:53900232	COSMIC_MUTATION	COSMIC_MUTATION
rs59309543	19:53903072	A	G/A
COSV54734338	19:53903072	COSMIC_MUTATION	COSMIC_MUTATION

Class	Source	Conseq. Type
somatic SNV	COSMIC	splice acceptor variant
SNP	dbSNP	splice donor variant
somatic SNV	COSMIC	splice donor variant
SNP	dbSNP	splice donor variant
somatic SNV	COSMIC	splice donor variant
somatic SNV	COSMIC	splice donor variant
somatic SNV	COSMIC	splice donor variant
SNP	dbSNP	splice donor variant
SNP	dbSNP	splice donor variant
SNP	dbSNP	splice donor variant
somatic SNV	COSMIC	splice donor variant
somatic SNV	COSMIC	splice donor variant
SNP	dbSNP	splice acceptor variant
somatic SNV	COSMIC	splice acceptor variant
somatic SNV	COSMIC	splice acceptor variant
somatic SNV	COSMIC	splice acceptor variant
SNP	dbSNP	splice acceptor variant
somatic SNV	COSMIC	splice acceptor variant
SNP	dbSNP	splice acceptor variant
somatic SNV	COSMIC	splice acceptor variant
